# Supplementary material for: Sentinel Lymph Node Biopsy in Atypical Spitz Tumor: A Systematic Review
Source: J Clin Med. 2024 May 30;13(11):3232. doi: 10.3390/jcm13113232 (PMC11172847; doi:10.3390/jcm13113232)
Supplement: Supplementary file 1 [file jcm-13-03232-s001.zip › jcm-2947524-Supplementary Material.pdf]

Supplementary Table S1. Definition of atypical Spitz tumor (AST) in the included studies.

First Author	Year	How AST Was Defined in the Paper
De Giorgi V	2022	AST definition in the clinical database [24]
Harms P.W.	2016	"Findings concerning for, but not fully diagnostic of, melanoma. Features vary by case, and may include: deep dermal extension, hypercellularity (cells in tumor nodules lack intervening collagen), cytologic atypia, increased mitoses (greater than rare), incomplete maturation" [10]
Massi D	2015	According to Barnhill et al. 2006 [13,25]
Batra S	2015	"Asymmetry, epidermal effacement, high cellular density, cytological atypia, high nuclear/cytoplasmic ration, mitotic rate > 2/mm^2" [26]
Busam KJ	2014	AST definition in the clinical charts [27]
Hung T	2013	Abnormalities of tumor cell organization; proliferation criteria; cytologic features [28]
Shen L	2013	AST definition in the clinical database [29]
Mills OL	2012	AST definition in the clinical database [30]
Caracò C	2012	"Size smaller than 10 mm; Breslow thickness > 1.0 mm; asymmetry, considering lesion central axis and defining two half; ulceration; circumscription; lateral pagetoid spread; maturation; deep pigmentation; presence of Kamino bodies; and mitotic activity; other minor histological criteria (such as epidermal thickening/thinning, hypergranulosis, kamino bodies, pagetoid spread, large epithelioid and/or spindle cells, regression, lymphovascolar invasion, perineural invasion, and deep pigmentation)" [31]
Raskin L	2011	According to Barnhill et al. 2006 [13,32]
Massi D	2011	According to Barnhill et al. 2006 [9,13]
Barnhill RL	2011	"Diameter ≥ 1 cm, surface ulceration, asymmetry, effacement of the epidermis (consumption), diminished or absent maturation, nodule formation, involvement of the subcutaneous fat (level V), dermal mitotic rate > 2 per square millimeter, deep or marginal mitoses, and cytological atypia beyond that acceptable for a Spitz tumor" [33]
Sepehr A	2011	AST definition in the clinical database (any form of atypia mentioned in the diagnosis of Spitz tumor) [19]
Cesinaro AM	2010	"Minimum of 1 mm thickness and the occurrence of at least one of the following nine histopathological features, selected from the literature because of their high frequency in atypical spitzoid lesions and melanomas, compared with Spitz's nevi: asymmetry, presence of ulceration, consumption of the epidermis, confluence of nests, solid growth, pushing-type growth, lack of maturation, prominent nucleoli1 and deep dermal mitoses" [34]
Ghazi B	2010	AST definition in clinical database [35]
Ludgate MW	2009	AST definition in clinical database [36]
Busam KJ	2009	AST definition in clinical database [37]
Murali R	2008	"Maximal dimension; tumor thickness (Breslow thickness in millimeters); Clark level; symmetry; presence or absence of Kamino bodies, hypergranulosis, epidermal thickening and/or thinning; junctional clefts; presence or absence of ulceration; presence or absence of pigment; cell types (spindle and/or epithelioid); dermal mitotic rate per square millimeter; and the presence of deep (within < .25 mm of the deep margin of the tumor) and abnormal/atypical mitoses. The presence or absence of expansile dermal nodules, marked nuclear and nucleolar pleomorphism, regressive changes (lymphoid infiltrates and angiofibrosis), lymphovascular invasion, and perineural invasion was noted. The presence of Pagetoid intraepidermal spread, which was separated into single cells or nests and classified as centrally and/or peripherally located within the tumor, was also evaluated." [38]

Urso C	2006	"Histological features characteristic of Spitz nevus mixed to histological features generally referred to malignant melanoma, appearing as spindle and/or epithelioid cell lesion deviating more or less from the stereotypical morphology of classic Spitz nevi; the tumor had not a clear-cut diagnosis of benign Spitz nevus or malignant melanoma" [39]
Gamblin TC	2006	AST definition in clinical database [40]
Su LD	2003	"Atypical spitzoid melanocytic proliferations that fell within any of several designations including atypical Spitz tumor, markedly atypical Spitz nevus, atypical epithelioid melanocytic proliferation of uncertain biologic potential, or borderline epithelioid melanocytic neoplasm"; melanoma with spitzoid features with discordant diagnosis among expert dermatopathologists; "patients in whom the diagnosis of melanoma could not be excluded" [41]
Lohmann CM	2002	AST definition in clinical database [15]

Supplementary Table S2. Molecular analysis in the included studies.

First Author	Year	Molecular Analysis
De Giorgi V	2022	Unclear [24]
Harms P.W.	2016	FISH (all patients) *Copy number aberration of CDKN2A.*[10]
Massi D	2015	FISH(some patients) *Heterozygous 9p21 loss, homozygous 9p21 loss, heterozygous 9p21 deletion.* [25]
Batra S	2015	FISH, aCGH (some patients) *Gains in 6p25 or 11q13 and homozygous deletions in 9p21.* [26]
Busam KJ	2014	FISH, aCGH (all patients) *Copy number changes of 6p, 6q, 9p, or 11q.* [27]
Hung T	2013	No [28]
Shen L	2013	FISH (all patients) *Copy number deletions in 6q23.* [29]
Mills OL	2012	No [30]
Caracò C	2012	No [31]
Raskin L	2011	FISH, aCGH (all patients) *Copy number changes of 6 and 11q.* [32]
Massi D	2011	FISH (all patients) *Three locus-specific identifier (RREB1, MYB, and CCND1) genes.* [9]
Barnhill RL	2011	No [33]
Sepehr A	2011	No [19]
Cesinaro AM	2010	FISH, MLPA (all patients) *Deletion of 9p21.* [34]
Ghazi B	2010	No [35]
Ludgate MW	2009	No [36]
Busam KJ	2009	No [37]
Murali R	2008	No [38]
Urso C	2006	No [39]
Gamblin TC	2006	No [40]
Su LD	2003	No [41]
Lohmann CM	2002	No [15]
